# BCG hydrogel promotes CTSS-mediated antigen processing and presentation, thereby suppressing metastasis and prolonging survival in melanoma

**DOI:** 10.1136/jitc-2021-004133

**Published:** 2022-06-22

**Authors:** Mirela Kremenovic, Alfred A Chan, Bing Feng, Lukas Bäriswyl, Steve Robatel, Thomas Gruber, Li Tang, Delphine J Lee, Mirjam Schenk

**Affiliations:** 1 Experimental Pathology, University of Bern Institute of Pathology, Bern, Switzerland; 2 Graduate School Cellular and Biomedical Sciences, University of Bern, Bern, Switzerland; 3 Division of Dermatology, Department of Medicine, The Lundquist Institute, Torrance, California, USA; 4 Institute of Bioengineering and Institute of Materials Science and Engineering, École Polytechnique Fédérale de Lausanne, Lausanne, VD, Switzerland

**Keywords:** melanoma, immunotherapy, macrophages, dendritic cells, antigen presentation

## Abstract

**Background:**

The use of intralesional *Mycobacterium bovis* BCG (intralesional live BCG) for the treatment of metastatic melanoma resulted in regression of directly injected, and occasionally of distal lesions. However, intralesional-BCG is less effective in patients with visceral metastases and did not significantly improve overall survival.

**Methods:**

We generated a novel BCG lysate and developed it into a thermosensitive PLGA-PEG-PLGA hydrogel (BCG hydrogel), which was injected adjacent to the tumor to assess its antitumor effect in syngeneic tumor models (B16F10, MC38). The effect of BCG hydrogel treatment on contralateral tumors, lung metastases, and survival was assessed to evaluate systemic long-term efficacy. Gene expression profiles of tumor-infiltrating immune cells and of tumor-draining lymph nodes from BCG hydrogel-treated mice were analyzed by single-cell RNA sequencing (scRNA-seq) and CD8^+^ T cell receptor (TCR) repertoire diversity was assessed by TCR-sequencing. To confirm the mechanistic findings, RNA-seq data of biopsies obtained from in-transit cutaneous metastases of patients with melanoma who had received intralesional-BCG therapy were analyzed.

**Results:**

Here, we show that BCG lysate exhibits enhanced antitumor efficacy compared to live mycobacteria and promotes a proinflammatory tumor microenvironment and M1 macrophage (MΦ) polarization in vivo. The underlying mechanisms of BCG lysate-mediated tumor immunity are dependent on MΦ and dendritic cells (DCs). BCG hydrogel treatment induced systemic immunity in melanoma-bearing mice with suppression of lung metastases and improved survival. Furthermore, BCG hydrogel promoted cathepsin S (CTSS) activity in MΦ and DCs, resulting in enhanced antigen processing and presentation of tumor-associated antigens. Finally, BCG hydrogel treatment was associated with increased frequencies of melanoma-reactive CD8^+^ T cells. In human patients with melanoma, intralesional-BCG treatment was associated with enhanced M1 MΦ, mature DC, antigen processing and presentation, as well as with increased CTSS expression which positively correlated with patient survival.

**Conclusions:**

These findings provide mechanistic insights as well as rationale for the clinical translation of BCG hydrogel as cancer immunotherapy to overcome the current limitations of immunotherapies for the treatment of patients with melanoma.

**WHAT IS ALREADY KNOWN ON THIS TOPIC**

- Intralesional BCG has been used in the treatment of cutaneous metastatic melanoma, resulting in regression of directly injected, and occasionally of distal lesions.
- However, intralesional BCG is less effective in patients with visceral metastases and did not significantly improve overall survival.

**WHAT THIS STUDY ADDS**

- Here, we demonstrate that BCG lysate is more effective than viable BCG in controlling tumor growth, inducing a T cell inflamed, proinflammatory tumor microenvironment, and promoting tumor immunity in melanoma.
- Moreover, we developed a novel BCG lysate-containing thermosensitive hydrogel that significantly prolongs survival and suppresses pulmonary metastases in murine melanoma.

**HOW THIS STUDY MIGHT AFFECT RESEARCH, PRACTICE AND/OR POLICY**

- Overall, our data provide rationale for the clinical implementation of BCG hydrogel as an effective and safe immunotherapeutic option to reduce the metastatic burden and prolong survival of patients with melanoma.

## Introduction

Despite significant breakthroughs in immunotherapy, cutaneous melanoma remains the most aggressive form of skin cancer with rising incidence and mortality rates.^1^ Although immunotherapeutics have remarkably improved the clinical outcomes of patients with cancer, a large proportion of patients are refractory to these treatment modalities and often relapse after initial tumor regression.^2^ Mycobacteria, such as the attenuated *Mycobacterium bovis* strain BCG, have been used as adjuvants to tumor vaccines, immunotherapy and chemotherapy, or intralesionally as neoadjuvant therapy to treat melanoma.^3 4^ Immunogenic components of BCG have been shown to induce strong immune responses through the activation of pattern recognition receptors (PRRs), including toll-like receptors (TLR) and NOD-like receptors (NLR) on innate immune cells such as macrophages (MΦ) and dendritic cells (DC).^5 6^ However, the precise mechanisms by which MΦ and DCs contribute to the BCG-mediated immune response against melanoma remain poorly understood. The use of intralesional BCG immunotherapy in melanoma dates back more than four decades and has demonstrated effective regression of directly injected and occasionally distant untreated lesions.^7^ While patients with solely cutaneous metastases were more likely to respond to intralesional BCG therapy, only a trend towards improved survival was observed,^8^ whereas patients with visceral metastases showed a lower incidence of tumor regression and no long-term survival benefits.^9^ Hence, despite being previously recommended as a possible intralesional therapy option for intransit melanoma in clinical practice guidelines,^10^ its use was largely discontinued, encouraging further development of novel, more effective mycobacteria-derived immunotherapies.

Local drug delivery approaches such as hydrogels, nanoparticles, rods and fibers have been widely investigated, as they not only reduce the toxicity of systemic therapies but also lessen the required amount to maintain therapeutic levels.^11^ Among these, hydrogels have gained much attention as they can be injected less invasively adjacent to the tumor providing controlled release of therapeutic agents. Hydrogels are a network of hydrophilic polymers, that can retain large amounts of water, or peptide/protein drugs, while still maintaining their physical structure.^12^ Moreover, hydrogels deliver drugs at predetermined rates for a defined period of time and protect the drug from the physiological environment, including changes in pH or enzymatic activity, thus improving bioavailability.^13^ Thermosensitive hydrogels undergo reversible thermal gelation in response to physiological body temperature. The synthetic copolymer poly(lactide-co-glycolide)-block-poly(ethylene glycol)-block-poly(lactide-co-glycolide) (PLGA-PEG-PLGA), whose PLGA and PEG components meet Food and Drug Administration (FDA)’s approval for clinical applications, is among the most commonly injected thermosensitive hydrogels used for local drug delivery.^13^

In this study, we investigated the efficacy of intratumoral BCG lysate treatment in murine melanoma models and compared it to live BCG. BCG lysate was more potent at inducing an immune-inflamed, proinflammatory tumor microenvironment (TME). The application of BCG lysate in the form of a thermosensitive hydrogel injected adjacent to the tumor promoted systemic immunity with immunological memory, reduced metastasis, and prolonged survival of tumor-bearing mice. Moreover, we show that BCG hydrogel enhanced Cathepsin S (CTSS) expression and antigen presentation, which was also observed in intralesional BCG treated patients and correlated to improved survival in patients with melanoma.

## Materials and methods

### Tissue culture

B16F10 melanoma cell line was purchased from American Type Culture Collection (ATCC). The MC38 colon adenocarcinoma cell line was provided by A. Zippelius (Department of Biomedicine, University of Basel, Basel, Switzerland). Murine cell lines, primary murine cells and human peripheral blood mononuclear cells (PBMCs) were cultured in complete Roswell Park Memorial Institute (RPMI)-1640 medium (Sigma; supplemented with 10% fetal bovine serum (FBS), 100 units/mL penicillin, 100 µg/mL streptomycin, 1 mM sodium pyruvate, and 2 mM L-glutamine). All cell lines tested negative for mycoplasma.

### Materials and reagents

BCG OncoTICE (MSD Merck Sharp & Dohme AG) was purchased at the Hospital Pharmacy, Inselspital Bern, Switzerland. Lyophilized bacteria were reconstituted in PBS according to the manufacturer’s protocol. BCG lysate was produced through ultrasonication of reconstituted bacteria for 10 min on ice (20 kHz, 30 s OFF and 30 s ON) using the Sonopuls (Bandelin). Protein concentration was measured by BCA protein assay (Thermo Fisher). PLGA-PEG-PLGA tri-block copolymers were synthesized by ring-opening copolymerization of lactide and glycolide in the presence of PEG as the initiator and Sn(Oct)2 as the catalyst. Briefly, anhydrous PEG1.5k (3 g, 2 mmol), lactide (4.66 g, 32.33 mmol) and glycolide (0.94 g, 8.1 mmol) were dissolved in anhydrous toluene (20 mL) at 100°C for 30 min and then catalytic Sn(Oct)2 was added to initiate the reaction. The reaction was kept for 24 hours at 100°C in the argon protection environment following precipitation in diethyl ether (1:20 v/v). The crude product was then purified by washing thrice with water (80°C). Residual water was removed by freeze drying to obtain tri-block copolymer; 20 µL of BCG or PBS was added to 100 µL polymer solution and vortexed at room temperature.

### Generation and activation of BMDM/BMDC and T cell proliferation assays

Murine bone marrow dendritic cells (BMDCs) and bone marrow derived MΦ (BMDMs) were generated from bone marrow of femurs and tibias of C57BL/6J (B6) mice and cultured in complete RPMI-1640 containing 10 ng/mL granulocyte-macrophage colony-stimulating factor (GM-CSF) (Sigma) or macrophage colony-stimulating factor (M-CSF) (20% L929 supernatant), respectively. Media including growth factors was replaced on day 4. On day 7, cells were harvested, and BMDCs were purified using magnetic anti-CD11c microbeads (Miltenyi Biotec). Cells were matured with 10 µg/mL BCG lysate for 48 hours before pulsing with 1 mg/mL ovalbumin (OVA) (EndoGrade, InvivoGen) for 24 hours. Subsequently, CD8^+^ T cells were purified from the spleens of OT-I mice using EasySep Mouse CD8^+^ T cell Isolation Kit (negative selection, STEMCELL Technologies) and co-cultured with matured BMDM or BMDC. T cell proliferation was assessed after 48 hours using the BrdU Cell Proliferation Assay Kit (BioVision).

### Monocyte isolation and measurement of CTSS activity

Human blood was obtained from healthy volunteers (Interregionale Blutspende SRK). PBMCs were isolated using Ficoll (GE Healthcare) and enriched for monocytes using the EasySep Human Monocyte Enrichment Kit without CD16 depletion (STEMCELL Technologies). Monocytes were rested for 24 hours before activation with 10 µg/mL BCG lysate or left untreated for 48 hours. CTSS activity was determined using the CTSS activity assay kit (Abcam) according to manufacturer’s protocol.

### Mice, tumor cell injection, and *in vivo* studies

C57BL/6 mice were purchased from Janvier Labs (France). *Batf3*-deficient (*Batf3^–/–^*) mice on a C57BL/6 background,^14^ were obtained from M. Suter (Department of Research, Bavarian Nordic GmbH, Martinsried, Germany; University of Zurich, Zurich, Switzerland). MHC-class I-restricted OVA-specific TCR (OT-I)–transgenic mice (TgTcraTcrb)1100Mjb,^15^ were obtained from University of Zürich (Animal Management System – iRATS). Nur77-GFP mice (Tg(Nr4a1-EGFP/cre)820Khog/J) were obtained from S. Freigang (Institute of Pathology, University of Bern). Age-matched and sex-matched 8–12 weeks old animals were used for all experiments. Mice were randomly assigned to different treatment groups prior to tumor injection.

B16F10 melanoma and MC38 colon cancer cells were engrafted subcutaneously (2×10^5^ cells) into the left flank on day 0. For some experiments, a second contralateral tumor was injected subcutaneously (5×10^5^ cells) on day 5. For lung metastases, 2×10^5^ cells were injected intravenously on day 5 post subcutaneous tumor injection. After randomization, mice were treated with intratumoral injections of BCG lysate (10 µg/mouse) or phosphate-buffered saline (PBS) into the primary tumor on days 7 and 11 or with BCG hydrogel (20 µg/mouse) or PBS hydrogel injection (subcutaneously) adjacent to the tumor on day 7. Contralateral tumors were left untreated. In all experiments mice were euthanized between days 12 and 18 after tumor inoculation or when tumor volume exceeded 1000 mm^3^. For MΦ depletion: anti-CSF1R antibody (500 µg, clone AFS98, Bio X Cell, BE0213) was injected intraperitoneally every other day starting 5 days prior to tumor injection. Tumor volume was assessed in two dimensions using a digital caliper in a blinded manner and calculated as follows: V = (length × width^2^)/2. Lungs were isolated on day 18 post tumor injection and numbers of metastases was assessed by visual counting. Lungs were embedded in paraffin and counterstained using H&E. Whole-slide images were acquired using a Pannoramic 250 Flash II (3D Histech). All mice were housed in specific pathogen-free conditions in the Central Animal Facility of the University of Bern.

### Nanostring mRNA profiling and cytokine/chemokine bead array

B16F10 melanomas were established and treated as described above. On day 12 tumors and peripheral blood samples (cardiac puncture) were collected. For NanoString profiling, tumors were homogenized in trizol (Sigma) followed by RNA extraction using RNeasy Mini Spin Columns (QIAGEN) according to the manufacturer’s protocol. RNA concentrations of all samples were normalized and analyzed using the Mouse Immunology Panel and the nCounter Digital Analyzer (NanoString Technologies). Data quality control, normalization and analysis were performed by nSolver V.4.0. Differential gene expression was analyzed using the *edgeR* and *Rtsne* packages in R. For cytokine/chemokine array, tumors were homogenized in a lysis buffer (4 µL/mg) consisting of PBS and 0.05% Tween-20, leupeptin (100 µM), aprotinin (10 µg/mL) and phenylmethylsulfonyl fluoride (PMSF) (200 µM) using the QIAGEN TissueLyser II. Tumor lysates were centrifuged at 10 000 g for 10 min at 4°C. Protein concentrations were measured using the bicinchoninic acid (BCA) protein assay kit (Thermo Fisher) and normalized to 5 mg/mL. Sera were obtained from blood by centrifugation in becton dickinson (BD) Microtainer blood collection tubes (BD SST). Samples were submitted to Eve Technologies for mouse cytokine array/chemokine array 44-plex analysis. Protein concentration with a log_2_(pg/mL) cut-off >2.5 was used for analysis with the *heatmap3* package in R and GraphPad Prism V.9.

### Flow cytometric analyses and cell sorting

Tumors were established as described above and harvested on day 14, mechanically dissociated and filtered through a 40 µM strainer (Thermo Fisher Scientific). The following antibodies against mouse antigens were used: anti-CD45.2 (104, BioLegend, 109830), anti-CD3ε (145–2 C11, BioLegend, 100306), anti-CD3 (17A2, BioLegend, 100236), anti-CD8α (53–6.7, BioLegend, 100730), anti-CD4 (RM4-5, BioLegend, 100538), anti-CD11c (N418, BioLegend, 117336), anti-CD11b (M1/70, BioLegend, 101236), anti-F4/80 (BM8, BioLegend, 123130), anti-NK1.1 (PK136, BioLegend,108708), anti-CD44 (IM7, BioLegend, 103047), anti-I-A/I-E (major histocompatibility complex (MHC) class II (MHCII), M5/114.15.2, BioLegend, 107614), anti-H-2Kb (MHC class I, AF6-88.5, BioLegend, 116518), anti-CD40 (3/23, BioLegend, 124624), anti-CD80 (16–10 A1, BioLegend, 104708), anti-CD83 (Michel-19, BioLegend, 121518), anti-CD86 (GL-1, BioLegend, 105006), Trp2 tetramers (iTAg Tetramer/PE H-2K^b^ tyrosinase-related protein (TRP)-2, MBL), anti–interferon-gamma (IFN-γ)(XMG1.2, BioLegend, 505830), anti-inducible nitric oxide synthase (iNOS) (CXNFT, Thermo Fisher, 46-5920-82) and anti-Arg1 (A1exF5, Thermo Fisher, 25-3697-82). Zombie Aqua Fixable Viability Kit (BioLegend, 423102) was used to discriminate dead cells. Fc receptor blocking was performed with anti-mouse CD16/32 (2.4G2, generated inhouse). Subsequently, cell surface markers were stained with antibodies in fluorescence-activated cell sorting (FACS) buffer (PBS with 2% FBS and 1 mM EDTA) for 30 min on ice. Intracellular staining for Arg1, iNOS and IFN-γ was performed using the eBioscience Foxp3/transcription factor staining buffer set following the manufacturer’s protocol. Samples were acquired using a CytoFLEX S flow cytometer (Beckman Coulter) and analyzed using FlowJo (Tree Star).

### Single-cell RNA sequencing

CD45^+^ cells for scRNA-seq were FACS purified from tumors and tumor-draining lymph node (TDLN) (ipsilateral, inguinal) on Moflo Astrios EQ cell sorter (Beckman Coulter). Library preparation was done according to the 10X Genomics protocols (Chromium Single-cell 3′ GEM Library and Gel Bead Kit v3) and sequenced on NovaSeq 6000 (Illumina SP flow cell, 100 bp paired-end reads). FASTQ files were generated using bcl2fastq V.2.20.0.422 from Illumina. Using Cellranger V.3.1.0, reads were aligned to the mouse genome mm10 from ENSEMBL GRCm38. Calculations were performed on the high performance computing (HPC) cluster of the University of Bern (UBELIX: http://www.id.unibe.ch/hpc, accessed on November 20, 2020). Cells >15% mitochondrial transcripts, and <1500 total molecule counts and <750 features were filtered out, resulting in 42’699 cells that passed QC metrics and 17’456 genes in tumors, and 37’228 cells and 16’686 genes in TDLN, respectively. Normalization and downstream analysis were performed using the *Seurat* package in R, as previously described.^16^ Gene ontology (GO) term enrichment analyses was performed in Cytoscape using the ClueGO plug-in.^17^

### DNA isolation and TCRβ sequencing

Tumors and spleens were isolated 14 days after tumor injection followed by genomic DNA extraction using the DNeasy Blood and Tissue Kit (QIAGEN) according to manufacturer’s protocol. TCRβ sequencing was performed using the ImmunoSEQ survey level assay (Adaptive Biotechnologies). Sequencing data were analyzed using the ImmunoSEQ analyzer (Adaptive Biotechnologies) and the *Vegan* package in R.

### Data analysis

TCGA gene expression data and clinical information were obtained using GDCRNATools. Counts were normalized using *EdgeR* package in R. The intralesional BCG patient cohort was accrued and selected in the routine care of patients treated at John Wayne Cancer Institute. Patients who were identified to be treated with intralesional BCG were enrolled and followed prospectively in this observational study. Bulk tumor RNA-seq data from intralesional BCG injected versus uninjected lesions (BCG TICE; Study ID BCG_J 001),^18 19^ were scored for their association with M1 MΦ,^20^ mature DC,^21^ and antigen processing and presentation pathway (Kyoto Encyclopedia of Genes and Genomes (KEGG)),^22^ using the *Singscore* package in R. CTSS expression and Kaplan-Meier survival analysis was performed with the *Survminer* package in R.

### Statistics

Statistical analyses were performed using GraphPad Prism V.7.0 or R. Statistical significance was calculated with one-way analysis of variance (ANOVA) followed by Tukey’s multiple comparisons, or two-way ANOVA followed by Šidák’s multiple comparisons test or two-tailed Student’s *t*-test. All measurements were taken from individual samples; n=independent biological replicates; p≤0.05 (*), p≤0.01 (**), p≤0.001 (***), p≤0.0001 (****).

## Results

### BCG lysate controls tumor growth and promotes a proinflammatory TME enriched in T cells and M1 MΦ

**Figure 1:**
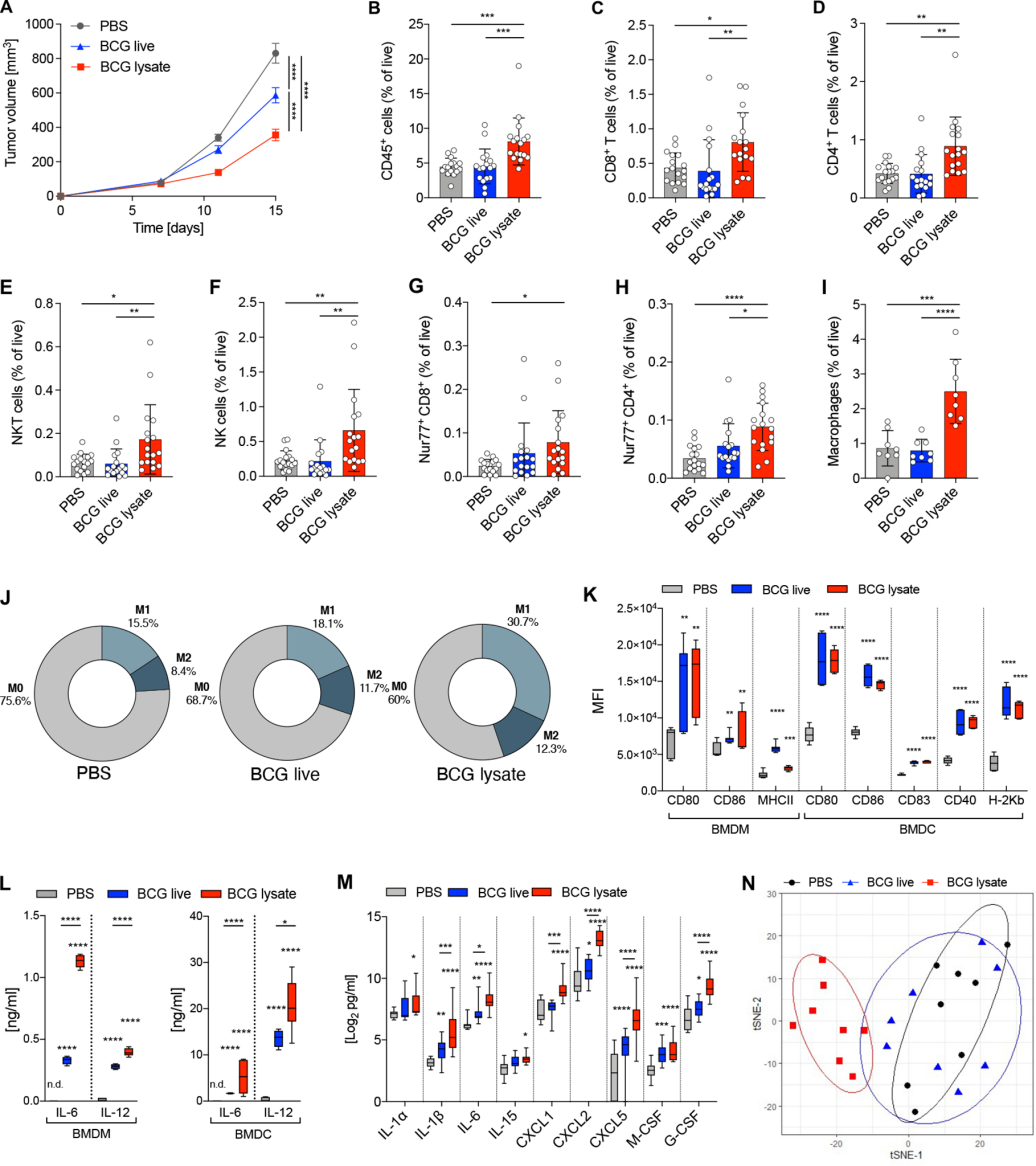
BCG lysate mediates tumor growth inhibition, immune cell-infiltration and a proinflammatory TME. (A) Growth curve of treated and untreated B16F10 melanoma. Data are represented as mean±SEM using two-way ANOVA followed by Šidák’s multiple comparisons test (n=25 per group, four independent experiments). (B–I) Relative frequencies of cell subsets measured by flow cytometry: (B) CD45^+^ cells, (C) CD8^+^ T cells, (D) CD4^+^ T cells, (E) NKT cells, (F) NK cells, (G) Nur77^+^ CD8^+^ T cells, (H) Nur77^+^ CD4^+^ T cells (n=17 per group, three independent experiments) and (I) MΦ (n=8 per group). Data are shown as mean±SD using one-way ANOVA, followed by Tukey’s multiple comparisons test. (J) Donut charts representing mean percentages of M0 MΦ, M1 MΦ and M2 MΦ within treated and untreated tumors. (K, L) BMDM and BMDC were stimulated or left untreated (media) for 48 hours. (K) Mean fluorescence intensity of maturation and activation markers as measured by flow cytometry (n=6–9 per group, three independent experiments). (L) Production of IL-6 and IL-12 measured by ELISA. Data are shown as box-and-whisker plot; the box extends between 25% and 75% and the whiskers extend to the minimum and maximum values, using two way-ANOVA followed by Šidák’s multiple comparisons test (n=4–7 per group, two independent experiments). (M) Protein expression levels of the indicated cytokines and chemokines from tumor lysates on day 12 post tumor injection (24 hours after last treatment). Data are shown as box-and-whisker plots; the box extends between 25% and 75% and the whiskers extend to the minimum and maximum values with a log_2_ cut-off >2.5, using two way-ANOVA followed by Šidák’s multiple comparisons test (n=10 per group). (N) Gene expression profiles of tumor lysates shown as ordination plots with 80% CI ellipses (n=8 per group). p≤0.05 (*), p≤0.01 (**), p≤0.001 (***), p≤0.0001 (****). ANOVA, analysis of variance; BMDC, bone marrow dendritic cell; BMDM, bone marrow derived microphages; MΦ, macrophage; TME, tumor microenvironment.

In view of the previously described studies showing a clinical benefit of intralesional BCG in the treatment of melanoma, we investigated the cellular and molecular mechanisms of BCG-mediated tumor immunity and compared the efficacy of previously used live BCG to a novel mycobacterial lysate.^4 23^ Therefore, we treated B16F10 melanoma-bearing mice with intratumoral injections of either live BCG or BCG lysate. Tumor growth was reduced in both treatment groups, however, BCG lysate was significantly more effective than live BCG (figure 1A). The frequencies and counts of specific immune cell subsets were determined by flow cytometry (figure 1B–I, online supplemental figure S2A–F), and the gating strategy for all immune cell subsets is shown (online supplemental figure S1). Tumors treated with BCG lysate showed increased frequencies of CD45^+^ leukocytes (figure 1B) and various lymphocyte subsets, including CD8^+^ and CD4^+^ T cells, natural killer T (NKT) and natural killer (NK) cells compared to tumors treated with live BCG or PBS (figure 1C–F). In addition, BCG lysate-treated tumors showed enhanced frequencies of activated Nur77-GFP^+^ (figure 1G, H) and antigen-experienced CD44^+^ CD8^+^ and CD4^+^ T cells, as well as IFN-γ^+^ CD8^+^ T cells (online supplemental figure S3A–C). Analysis of immune cell frequency at day 9 post tumor injection (2 days after the first treatment) already showed an increased frequency of immune cells in BCG lysate-treated tumors (online supplemental figure S4B), while no significant difference in tumor size was observed (online supplemental figure S4A). Finally, BCG lysate also significantly enhanced the frequency of tumor-associated MΦ (TAM, figure 1I) and increased the proportion of iNOS^+^ M1 MΦ, while the proportion of Arg1^+^ M2 MΦ was not significantly altered (figure 1J). Tumors that were treated with live BCG did not show any significant changes in the frequencies nor the proportions of TAM subsets (figure 1I, J). We next examined if BCG affects the activation state of both murine BMDMs and BMDCs in vitro. Our data show that both live and BCG lysate induced potent MΦ activation and DC maturation (figure 1K), as well as the secretion of proinflammatory cytokines (interleukin (IL) 6, IL-12), which was significantly higher in BCG lysate-stimulated BMDMs and BMDCs (figure 1L).

10.1136/jitc-2021-004133.supp1 Supplementary data

To investigate the BCG-induced changes within the TME more comprehensively, we analyzed the protein expression of 44 key cytokines and chemokines in tumors treated with live BCG or BCG lysate. Notably, tumors treated with BCG lysate showed significantly increased levels of proinflammatory cytokines (IL-1α/β, IL-6 and IL-15), CXCR2-binding chemokines produced by MΦ during inflammatory responses (CXCL1, CXCL2 and CXCL5),^24 25^ and growth factors (M-CSF and G-CSF), compared to tumors treated with live BCG or PBS (figure 1M). In contrast, no increase in inflammatory cytokines nor chemokines was detected in the sera of treated mice, indicating that systemic inflammation was not induced (online supplemental figure S5). Moreover, transcriptomic profiling of BCG lysate-treated tumors revealed a shift in the gene expression profile towards an immune-inflamed TME, whereas live BCG-treated tumors showed no clear separation from control tumors (figure 1N). Taken together, these data clearly demonstrate that BCG lysate is more potent than live BCG in controlling tumor growth, inducing proinflammatory cytokines and chemokines, and enhancing the infiltration of effector cells. Therefore, our subsequent studies to investigate the underlying cellular and molecular mechanisms focus on BCG lysate.

### Tumoricidal effects mediated by BCG lysate are dependent on TAM and cross-presentation by DC

**Figure 2:**
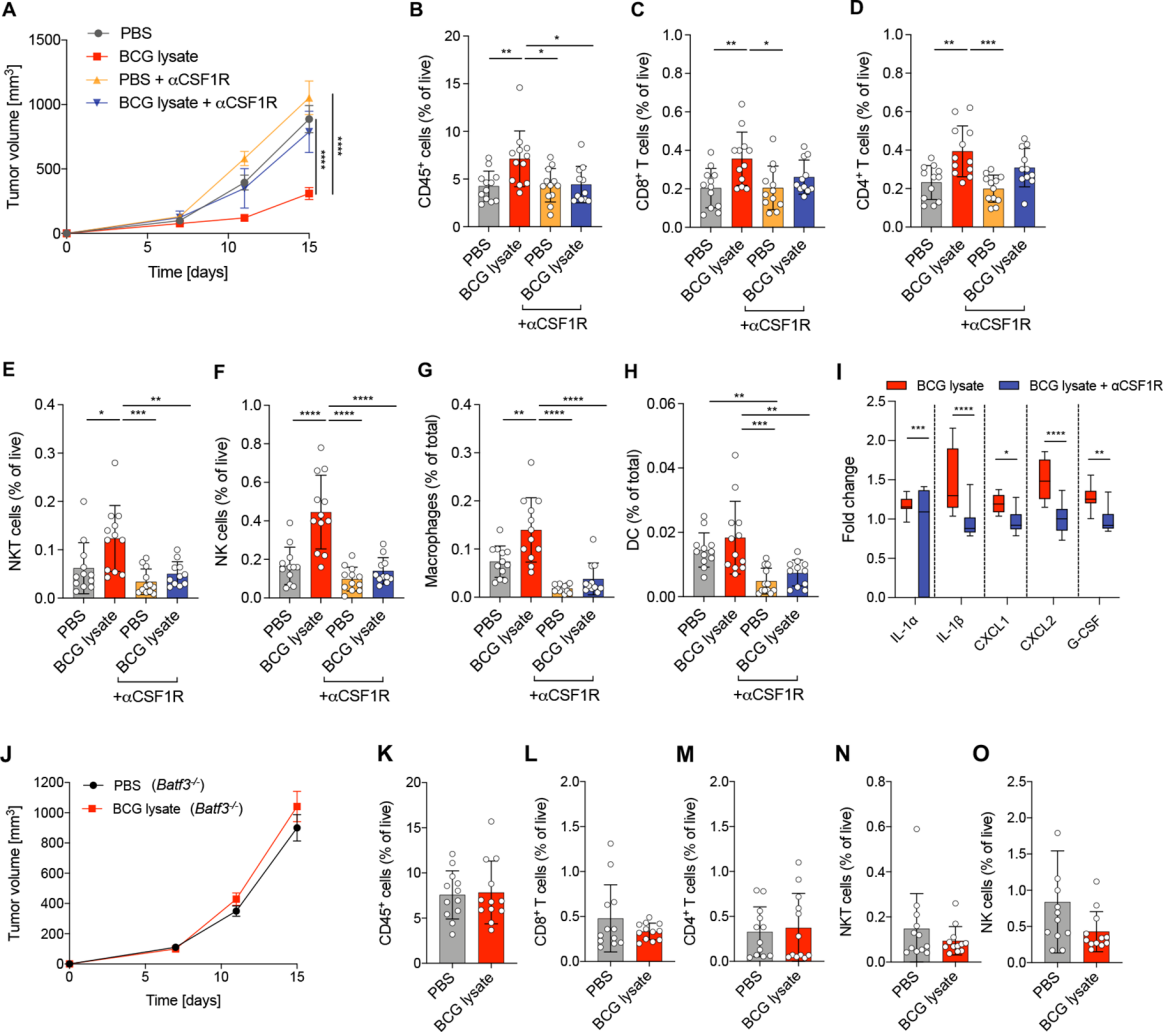
Tumoricidal effect of BCG lysate is dependent on TAM and DC. (A) Growth curve of treated and untreated B16F10 melanoma with and without MΦ depletion. Data are represented as mean±SEM using two-way-ANOVA followed by Šidák’s multiple comparisons test (n=12 per group except BCG +anti-CSF1R, n=11; two independent experiments). (B–H) Relative frequencies of cell subsets measured by flow cytometry: (B) CD45^+^ cells, (C) CD8^+^ T cells, (D) CD4^+^ T cells, (E) NKT cells, (F) NK cells, (G) MΦ and (H) DC. (I) BCG lysate-induced fold change of the indicated cytokines and chemokines from tumor lysates on day 12 after tumor injection. Data are shown as box-and-whisker plot; the box extends between 25% and 75% and the whiskers extend to the minimum and maximum values (BCG, n=11; BCG +anti-CSF1R, n=9; two independent experiments). Statistical significance was determined using two way-ANOVA followed by Šidák’s multiple comparisons test. (J) Growth curves of BCG lysate-treated or PBS-treated B16F10 tumors in *Batf3^–/–^* mice, represented as mean±SEM using two-way-ANOVA followed by Šidák’s multiple comparisons test (n=12 per group, two independent experiments). (K–O) Relative frequencies of cell subsets measured by flow cytometry: (K) CD45^+^ cells, (L) CD8^+^ T cells, (M) CD4^+^ T cells, (N) NKT cells and (O) NK cells. Data are represented as mean±SD using one-way ANOVA followed by Tukey’s multiple comparisons test (n=12 per group, two independent experiments). p≤0.05 (*), p≤0.01 (**), p≤0.001 (***), p≤0.0001 (****). ANOVA, analysis of variance; DC, dendritic cell; MΦ, macrophage; TAM, tumor-associated microphage.

Given the effect of BCG lysate on MΦ activation and DC maturation, we investigated whether BCG-induced antitumor immune responses were dependent on TAM and DC. Thus, we evaluated the effect of intratumoral BCG lysate treatment in MΦ-depleted mice using anti-CSF1R monoclonal antibody. Intriguingly, MΦ depletion abrogated the tumor growth control (figure 2A), as well as the increased frequencies of various immune cell subsets observed in BCG-treated, non-depleted mice, such as CD8^+^ and CD4^+^ T cells, NKT and NK cells, MΦ and DC (figure 2B–H). Depletion of MΦ with anti-CSF1R was confirmed in the spleens of mice, which showed significantly reduced frequencies of MΦ (>80% depletion, online supplemental figure S6A) and CD11c^+^ DC (>70% depletion, online supplemental figure S6B). In addition, the secretion of various BCG-induced proinflammatory cytokines and chemokines such as IL-1α, IL-1β, CXCL1 and CXCL2, as well as the growth factor G-CSF was significantly lower in MΦ-depleted mice (figure 2I). To assess the role of conventional DC (cDC1) in BCG-induced immune responses, we used *Batf3^-/-^* mice, lacking cross-presenting cDC1.^14^ Tumor growth control by BCG lysate was abrogated in *Batf3^-/-^* mice (figure 2J) and there was no BCG-induced increase in the frequencies of CD45^+^ immune cells, CD8^+^ and CD4^+^ T cells, NKT and NK cells (figure 2K–O).

### BCG hydrogel increased survival and decreased metastatic potential of B16F10 melanoma

**Figure 3:**
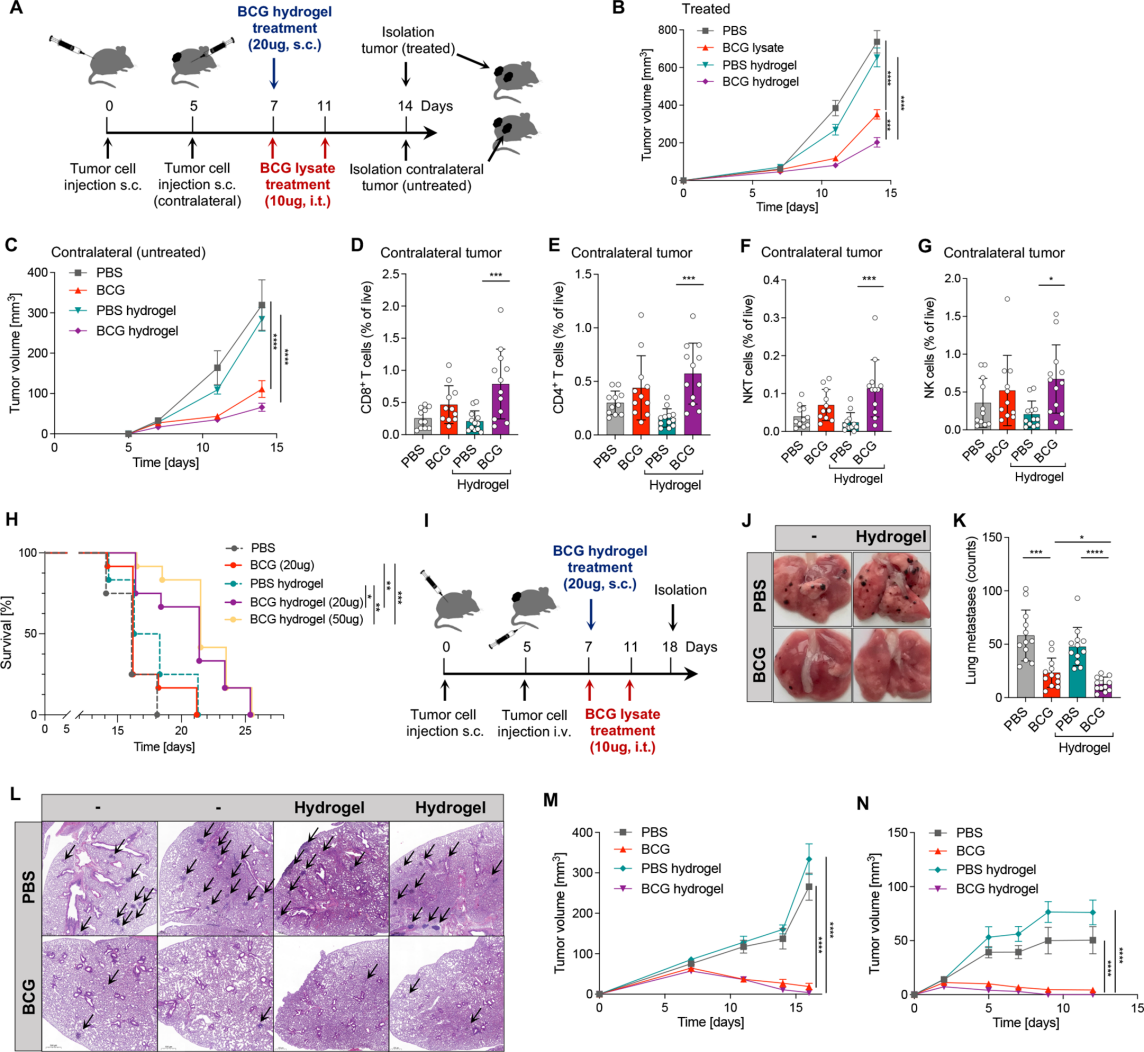
BCG hydrogel treatment prolonged survival and reduced the formation of lung metastases. (A) Scheme of the experimental set-up. (B, C) Growth curves of treated and contralateral (untreated) tumors. Data are represented as mean±SEM using two-way ANOVA followed by Šidák’s multiple comparisons test (n=12 per group, two independent experiments). (D–G) Relative frequencies of cell subsets measured by flow cytometry in contralateral tumors: (D) CD8^+^ T cells, (E) CD4^+^ T cells (F) NKT cells and (G) NK cells. Data are shown as mean±SD using one-way ANOVA, followed by Tukey’s multiple comparisons test (n=12 per group, two independent experiments). (H) Kaplan-Meier survival curves. Significance was determined by log-rank (Mantel-Cox) test (n=12 per group except PBS, n=4). (I) Experimental set-up scheme of lung metastasis model. (J) Representative images of B16F10 lung metastases on day 18 post tumor injection. (K) Visual quantification of the metastatic foci on lung surfaces. Data are represented as mean±SD (n=12 per group, two independent experiments). (L) Representative H&E staining of lung metastases. Black arrows indicate metastatic foci. (M) Growth curve of MC38 tumors treated with intratumoral BCG or BCG hydrogel. (N) Growth curve of tumors after re-challenge with MC38 cells. Data are represented as mean±SEM using two-way-ANOVA followed by Šidák’s multiple comparisons test (PBS, n=10; BCG, n=12; PBS hydrogel, n=6; BCG hydrogel, n=12; two independent experiments). p≤0.05 (*), p≤0.01 (**), p≤0.001 (***), p≤0.0001 (****). ANOVA, analysis of variance.

Thermosensitive hydrogels can serve as in situ deposits for sustained release of drugs and are widely used for local delivery of cancer therapies.^26^ To investigate the use of hydrogels as an effective delivery system for BCG, we loaded a PLGA-PEG-PLGA hydrogel with equivalent amounts of BCG lysate as used intratumorally and injected it subcutaneously djacent to the tumor on day 7 post tumor injection. A contralateral (untreated) tumor was injected 5 days after the primary tumor to assess the systemic effect of BCG hydrogel (figure 3A). PBS-loaded hydrogel was used as control. Strikingly, BCG hydrogel significantly delayed tumor growth of primary (figure 3B) as well as contralateral untreated tumors (figure 3C). Consistently, cell profiling of BCG hydrogel-treated primary tumor showed increased frequencies of CD45^+^ immune cells, CD8^+^ and CD4^+^ T cells, NKT cells and NK cells, as well as iNOS^+^ M1 MΦ (online supplemental figure S7A–G). Notably, increased frequencies of CD8^+^ and CD4^+^ T cells, as well as NKT and NK cells were observed in the untreated contralateral tumors of mice that received BCG hydrogel (figure 3D–G), indicating systemic immunity. These results prompted us to compare the effect of BCG hydrogel with continuous intratumoral injections of BCG lysate in long-term treatment and survival studies in mice inoculated with B16F10 melanoma. This revealed improved survival in mice treated with BCG hydrogel (median survival: 21 days) compared to BCG lysate (median survival: 16 days), which corresponds to a prolonged survival of 23.8% (HR: 0.4511, 95% CI 0.1901 to 1.071). No additional benefit was observed with a higher dose of BCG lysate in hydrogel (50 ug, figure 3H).

Since distant metastases are a leading cause of death in patients with advanced melanoma, we investigated the potential of BCG hydrogel to prevent experimental pulmonary metastases.^27^ Tumor cell injection and treatment were performed as shown in the scheme of the experimental set-up (figure 3I). Quantification of metastatic nodules showed suppression of pulmonary metastasis in BCG-treated mice, with significantly lower numbers in BCG hydrogel compared with intratumoral BCG lysate treatment (figure 3J, K). Histological examination of the lungs confirmed that the BCG-treated mice had few metastases, whereas the control treated mice had numerous metastatic nodules in the lung parenchyma (figure 3L). Given the significant antitumor effect of BCG hydrogel in murine melanoma, we further tested the application of BCG hydrogel in the immunocompetent murine colon adenocarcinoma model MC38. Tumors were induced by subcutaneous inoculation of MC38 colon adenocarcinoma cells and treated with BCG lysate or BCG hydrogel according to our previous treatment regimen. Complete regression of MC38 tumors was achieved by both intratumoral BCG lysate as well as BCG hydrogel implantation (figure 3M). These cured mice were protected from tumor growth on re-challenge with MC38 cells, whereas control mice developed tumors, indicating long-term and immunological memory, confirming an intact immune system capable of recall responses (figure 3N).

### BCG hydrogel treatment promotes antigen processing and presentation in MΦ and DC

**Figure 4:**
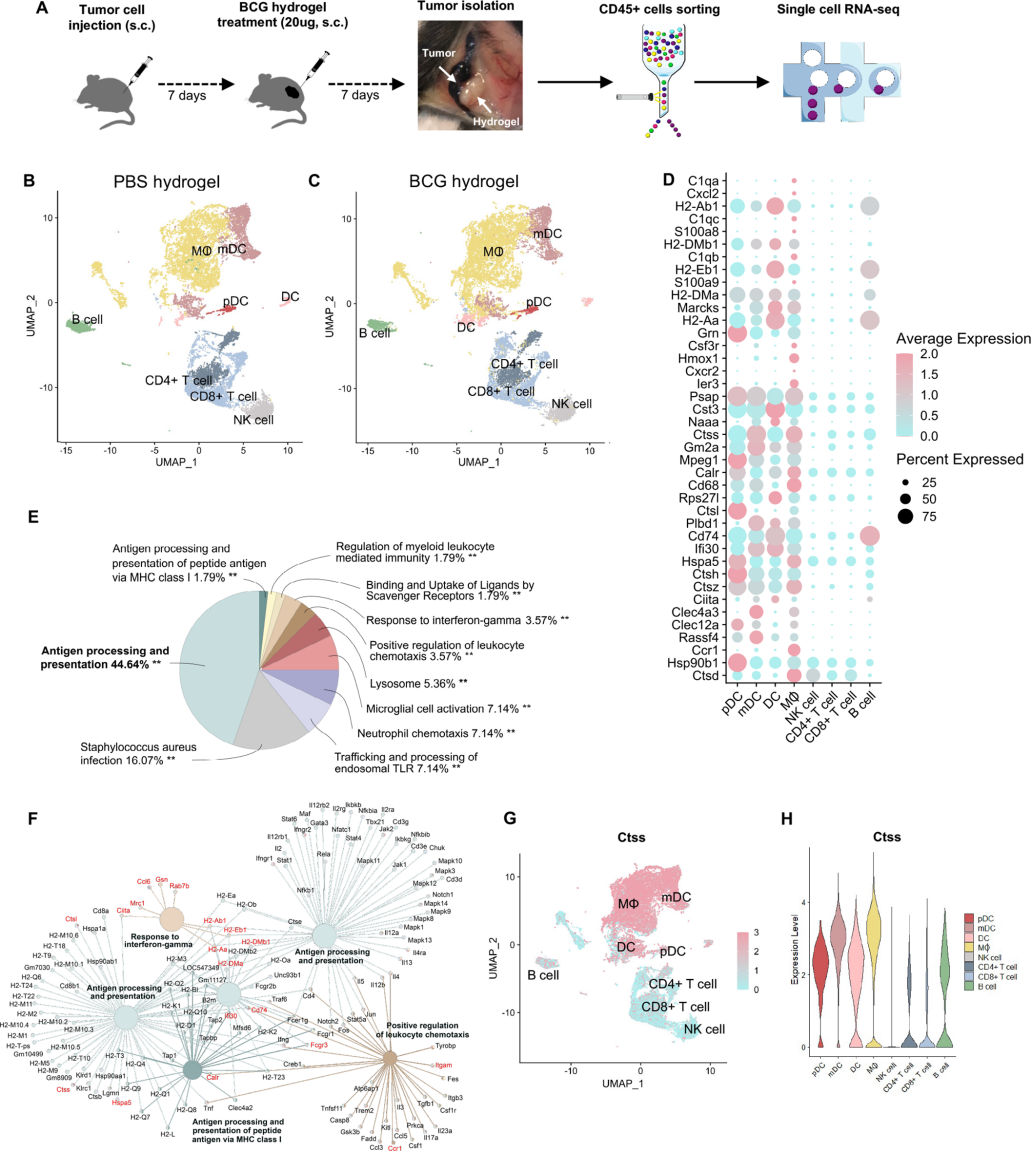
ScRNA-seq analysis of BCG hydrogel-treated tumors showed enhanced antigen processing and presentation in DC and MΦ. (A) Scheme of experimental workflow. (B, C) Uniform Manifold Approximation and Projection(UMAP) plot demonstrating clustering and cell type identities obtained for both treatment groups. Cluster annotations: B cell, CD4^+^ T cell, CD8^+^ T cell, DC, mDC, pDC, MΦ and NK cell. (D) Top 40 differentially upregulated genes between BCG hydrogel-treated and PBS hydrogel-treated tumors within identified cell types. Sorted from highest log_2_ fold change (top) to lowest (bottom), log_2_ fold change cut-off=0.25 and false discovery rate (FDR)=0.05. (E, F) Gene ontology analysis of biological processes for genes upregulated in BCG hydrogel-treated versus PBS hydrogel-treated tumors. (E) Pie charts demonstrating percentages of biological processes from upregulated genes and (F) net plots showing the relationships between genes associated with selected GO terms. (G) UMAP plot showing distribution of CTSS expression. (H) Violin plot demonstrating CTSS expression level in distinct cell populations. CTSS, Cathepsin S; DC, dendritic cell, GO, gene ontology; mDC, myeloid DC; MΦ, macrophage; pDC, plasmacytoid DC; scRNA, single-cell RNA.

To characterize the BCG-induced transcriptomic changes of specific immune cell subsets in B16F10 melanoma, we performed single-cell RNA sequencing (scRNA-seq) on CD45^+^ cells from BCG hydrogel-treated and PBS hydrogel-treated tumors and TDLN. CD45^+^ cells were purified using fluorescence-activated cell sorting and subsequently processed for scRNA-seq (figure 4A). Significantly overexpressed cluster makers were correlated to gene set libraries of the Enrichr database to identify corresponding cell types.^28^ In tumors treated with PBS hydrogel (figure 4B) or BCG hydrogel (figure 4C), eight major cell populations were identified: B cells, CD4^+^ T cells, CD8^+^ T cells, lymphoid DC, myeloid DC, plasmacytoid DC, MΦ and NK cells. Notably, the MΦ and DC clusters showed the most significant changes between BCG hydrogel and PBS hydrogel treatment. Differentially upregulated genes were primarily expressed within the MΦ and DC clusters, of which the top 40 are shown (figure 4D). Pathway analysis of the significantly upregulated genes revealed that these are predominantly involved in ‘antigen processing and presentation’ (figure 4E) and were associated with ‘response to IFN-γ’, ‘antigen processing and presentation via MH class I’, as well as the ‘positive regulation of leukocyte chemotaxis’ (figure 4F). The BCG hydrogel-induced genes include genes encoding major histocompatibility class II (H2-Ab1, H2-DMb1, H2-Dma, H2-Aa), MΦ markers (Cxcl2, Marcks, Cd68, Itgam, Ccl6, Fcgr3, Mpeg1, Ifi30), DC markers (Clec4a3, Clec12a), and several cathepsins (*Ctss, Ctsl, Ctsh, Ctsz, Ctsd*), with *Ctss* being most strongly induced. The cysteine protease CTSS is highly expressed in antigen-presenting cells (APC) and mediates the proteolytic processing of antigens and the cleavage of the invariant chain to allow subsequent binding of exogenous peptides to the MHC class II molecule for antigen presentation.^29^ Previous studies have demonstrated CTSS to be implicated in antigen processing inside phagosomes for transporter associated with antigen processing (TAP)-independent cross-presentation of antigens on MHC class I to CD8^+^ T cells.^30^ Consistently, *Ctss* was predominantly expressed within the MΦ and DC clusters in our data set (figure 4G, H). Comparison of scRNA-seq data of tumors treated with BCG hydrogel versus intratumoral BCG lysate showed major changes within the MΦ cluster (online supplemental figure S8A) with enhanced antigen presentation and processing pathways in tumors treated with BCG hydrogel (online supplemental figure S8B).

**Figure 5:**
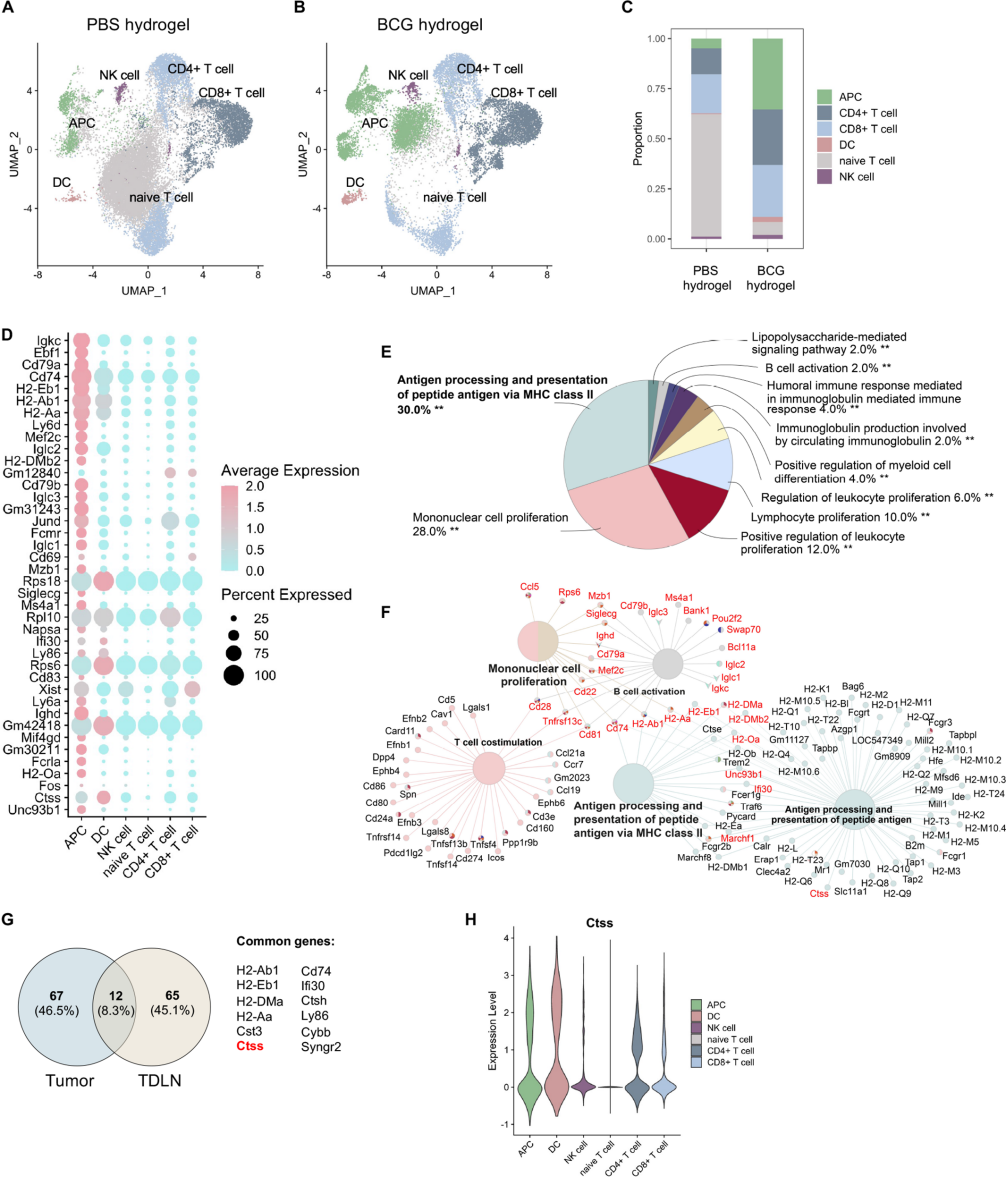
BCG hydrogel promoted antigen processing and presentation in TDLN. (A, B) UMAP plot demonstrating clustering and cell type identities obtained for both treatment groups. Cluster annotations: APC, DC, NK cell, naive T cell, CD4^+^ T cell, CD8^+^ T cell. (C) Bar plot demonstrating proportion of identified cell types across treatment groups. (D) Top 40 differentially upregulated genes between BCG hydrogel and PBS hydrogel treatment groups within identified cell types. Sorted from highest log_2_ fold change (top) to lowest (bottom), log_2_ fold change cut-off=0.25 and FDR=0.05. (E, F) GO analysis of biological processes for genes upregulated in BCG hydrogel-treated versus PBS hydrogel-treated TDLN. (E) Pie charts demonstrating percentages of biological processes from upregulated genes and (F) net plots showing the relationships between genes associated with selected GO terms. (G) Venn diagram of BCG hydrogel-induced genes in tumors and TDLN and common genes. (H) Violin plot demonstrating CTSS expression level within cell subsets. APC, antigen-presenting cell; CTSS, Cathepsin S; DC, dendritic cell; GO, gene ontology; TDLN, tumor-draining lymph node.

TDLN are crucial sites for priming T cells to trigger tumor-specific immune responses and systemic immunity.^31 32^ To investigate BCG hydrogel-induced systemic responses, CD45^+^ cells were sorted from TDLN of melanoma-bearing mice and subjected to scRNA-seq analysis. Clusters denoting APC, DC, NK cells, naïve T cells, CD8^+^ and CD4^+^ T cells were identified (figure 5A, B). Proportion analysis revealed an increase in APC, DC, CD4^+^ and CD8^+^ T cell clusters in the BCG hydrogel treated mice (figure 5C) and a decrease in naïve T cells (figure 5C), which was confirmed by flow cytometry analysis of activated T cells in the TDLN (online supplemental figure S9A).

Differentially upregulated genes in the BCG hydrogel treatment group were predominantly expressed in the APC and DC clusters, of which the top 40 are shown (figure 5D). Pathway analysis of the differentially expressed genes showed upregulation of ‘antigen processing and presentation’, as well as ‘mononuclear cell proliferation’ (figure 5E). Moreover, these genes were associated with ‘antigen processing and presentation via MH class II’ and ‘B cell activation and T cell co-stimulation’ (figure 5F). Comparison of BCG hydrogel-induced genes in tumors and TDLN revealed 12 common genes, which include *Ctss* (figure 5G). *Ctss* expression levels were highest within the APC and DC clusters, supporting the finding that BCG hydrogel promotes antigen processing and presentation via upregulation of CTSS (figure 5H).

### BCG induces CTSS expression which promotes antigen processing and presentation and correlates with improved survival in patients with melanoma

**Figure 6:**
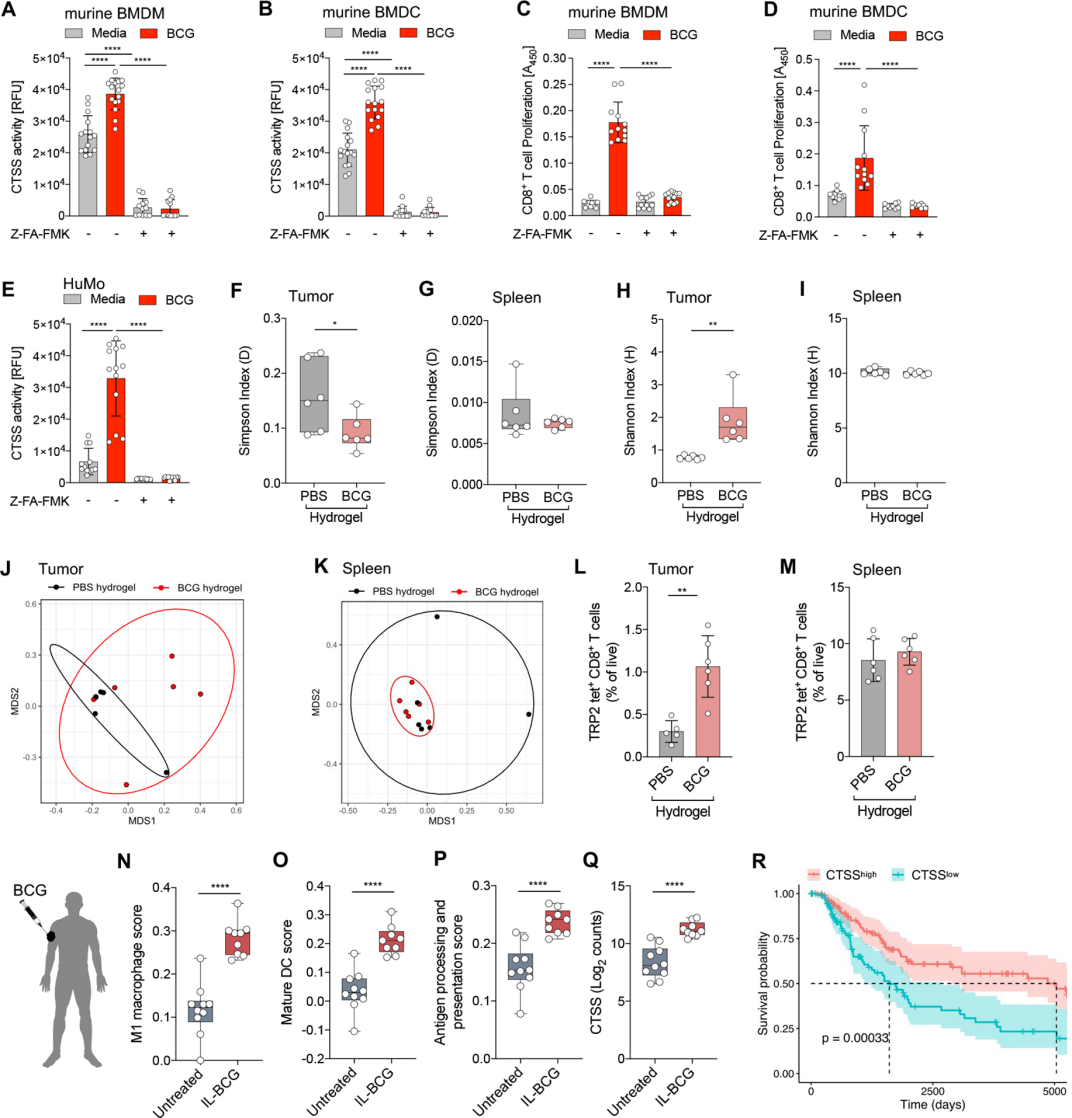
BCG-induced CTSS activity mediates antigen processing and presentation and is associated with improved survival in patients with melanoma. (A, B) CTSS activity in cell lysates from (A) BMDM and (B) BMDC (n=12–16 per group, two independent experiments). (C, D) Proliferation of OT-I cells co-cultured with BCG-stimulated and OVA-pulsed (C) murine BMDM or (D) BMDC. Data are shown as mean±SD using one-way ANOVA and followed by Tukey’s multiple comparisons test (n=9–12 per group, two independent experiments). (E) CTSS activity in cell lysates from huMo. Data are represented as mean±SD using one-way ANOVA and followed by Tukey’s multiple comparisons test (n=10–13 per group, four individual replicates, two independent experiments). (F, G) Simpson’s index of clonality in (F) tumor and (G) spleen as assessed by TCRß sequencing. (H, I) Shannon index of diversity in (H) tumor and (I) spleen. Data are represented as mean±SD using unpaired, two-tailed Student’s *t*-test (n=6 per group). (J, K) MDS ordination plots using Morisita-Horn dissimilarity distance metrics of TCR clones in (J) tumor and (K) spleen with 80% CI ellipses. (L, M) Relative frequencies of TRP-2 tetramer-positive CD8^+^ T cells, as a proportion of total CD8^+^ T cells in (L) tumor (PBS hydrogel, n=5; BCG hydrogel, n=6) and (M) spleen (n=6 per group). (N–Q) RNA-seq data from punch biopsies of BCG-injected tumors (n=9) and uninjected tumors (n=11) from patients with intransit melanoma cutaneous metastases. (N–P) Signature scores of (N) M1 MΦ, (O) mature DC and (P) antigen presentation pathway (KEGG) in BCG-injected and uninjected lesions. (Q) CTSS expression of BCG-injected and uninjected nodules shown as normalized, log_2_ transformed counts. Data are shown as box-and-whisker plots using two-tailed Student’s t-test. The box extends between 25% and 75%, and the whiskers extend to the minimum and maximum. (R) Kaplan-Meier survival curves for CTSS^high^ and CTSS^low^ SKCM patients (overall survival). p≤0.05 (*), p≤0.01 (**), p≤0.001 (***), p≤0.0001 (****). ANOVA, analysis of variance; BMDC, bone marrow dendritic cell; BMDM, bone marrow derived macrophage; CTSS, Cathepsin S; DC, dendritic cell; huMo, human monocytes; MDS, multidimensional scaling; MΦ, macrophage; SKCM, skin cutaneous melanoma; TCR, T cell receptor; TRP, tyrosinase-related protein.

Based on the crucial role of CTSS in antigen processing and (cross-)presentation, we assessed if BCG alters CTSS activity and thus the ability of APC to cross-present antigen.^30^ CTSS activity was significantly increased in BCG-stimulated BMDM (figure 6A) and BMDC (figure 6B) and was almost completely abrogated by the cysteine protease inhibitor Z-Phe-Ala fluoromethyl ketone (Z-FA-FMK; figure 6A, B).^33^ In addition, enhanced cross-presentation of exogenous OVA antigen was observed in BCG-stimulated BMDM (figure 6C) and BMDC (figure 6D) as measured by OT-I CD8^+^ T cell proliferation which was inhibited by blocking CTSS (figure 6C, D). Moreover, we found an approximately fourfold induction of the CTSS activity in BCG-stimulated human monocytes (huMo), which was almost completely abolished by inhibitor treatment (figure 6E). To assess if increased CTSS activity translates to improved antigen processing and presentation, we examined the TCR repertoire in tumors treated with BCG versus PBS hydrogel. Therefore, we performed TCR sequencing on CD8^+^ T cells from tumors and matched spleens isolated on day 7 post BCG hydrogel implantation. TCR sequencing revealed decreased TCR clonality in BCG compared to PBS hydrogel-treated tumors, as indicated by the Simpson’s index (figure 6F) and a higher diversity of the TCR repertoire in BCG hydrogel-treated tumors as indicated by the Shannon Index (figure 6H),^34^ whereas no significant differences were observed in the spleen (figure 6G, I). In addition, Shannon’s Indices of all tumors were smaller (<4.0) than those of the spleens (>9.0), suggesting that tumor-reactive T cell clones were selectively expanded in the tumor. The Morisita-Horn distance is used to assess TCR repertoire dissimilarities by quantifying the overlap between two repertoires.^35^ Multidimensional scaling of the Morisita-Horn distance showed a lower clonal overlap in BCG hydrogel-treated tumors (figure 6J), indicating a greater dissimilarity of TCR clones, however, in spleen a higher overlap was observed (figure 6K). These findings indicate increased richness and diversity of the T cell repertoire in BCG hydrogel-treated tumors. To determine whether BCG hydrogel treatment alters the frequency of tumor-specific CD8^+^ T cells we performed a tetramer stain using MHC class I tetrameric complexes bearing SVYDFFVWL peptides derived from TRP-2 (H-2kb TRP-2). TRP-2 is a human melanoma-specific antigen with an ortholog in murine B16F10 melanoma cells and TRP-2 specific CD8^+^ T cells have been described in both humans and mice.^36^ We found that tumors treated with BCG hydrogel showed enhanced frequencies of TRP-2-specific CD8^+^ T cells (figure 6L), while no significant change was observed in the spleen (figure 6M).

To investigate the clinical relevance of our findings we examined human melanoma treated with intralesional BCG. Therefore, we analyzed bulk RNA-seq data of biopsies obtained from intransit cutaneous metastases from patients with melanoma that received intralesional BCG (BCG TICE; Study ID BCG_J 001),^18 19^ for the association with M1 MΦ,^20^ and mature DC,^21^ using previously defined gene signatures (online supplemental table S1). BCG-injected tumors displayed significantly higher levels of M1 MΦ (figure 6N) and mature DC (figure 6O) compared with untreated lesions. Moreover, the signature score of genes involved in the antigen processing and presentation pathway (KEGG, online supplemental table S1) was enhanced in BCG-treated versus untreated lesions (figure 6P).^22^ In addition, CTSS expression was also increased in BCG-injected nodules, providing further evidence of BCG-induced CTSS activity (figure 6Q). Furthermore, CTSS expression positively correlated with the M1 MΦ and mature DC signature in the skin cutaneous melanoma (SKCM) cohort from The Cancer Genome Atlas (TCGA) consortium (online supplemental figures S10A, B).^37^ Stratification of the patients in CTSS^high^ and CTSS^low^ (split by quartile), revealed a significantly increased expression of the M1 MΦ and mature DC signature in CTSS^high^ patients (online supplemental figures S10C, D). Finally, Kaplan-Meier survival analysis between CTSS^high^ and CTSS^low^ patients with SKCM revealed a significantly prolonged overall survival of the CTSS^high^ patient group (HR: 0.4939, 95% CI 1.367 to 2.999; figure 6R).

## Discussion

In this study, we demonstrate that BCG lysate is more effective than live BCG to promote an immune-inflamed TME and tumor immunity in melanoma. Moreover, we developed a novel, BCG lysate-loaded, thermosensitive hydrogel and tested its application in preclinical murine melanoma models. BCG hydrogel significantly prolonged survival and suppressed pulmonary metastases in mice, suggesting its potential use as neoadjuvant therapy for the treatment of patients with metastatic melanoma. BCG hydrogel-mediated responses were largely dependent on MΦ and DC and associated with enhanced antigen processing and presentation pathways through CTSS. The clinical relevance of these findings is reflected in increased CTSS expression observed in patients with melanoma treated with intralesional BCG, and the positive correlation between CTSS expression and improved survival in patients with melanoma.

Increased frequencies of tumor-infiltrating lymphocytes (TIL) are generally a good prognostic factor in multiple solid tumors including melanoma and strategies to enhance immune cell infiltration can improve clinical outcomes.^38^ In this study, we demonstrate that BCG lysate is more effective than live BCG in recruiting TIL and promoting a chemokine-rich, proinflammatory TME while eliminating the risks associated with injecting live bacteria.^39^ Unlike purified antigens or cell-wall fragments, BCG lysate retains the complete antigenic profile and additionally contains released immunostimulatory intracellular molecules that act as PRR agonists, such as DNA or RNA fragments, explaining the increased potency.^40^

Furthermore, we show that BCG lysate-mediated antitumor immune responses depend on MΦ and DC, which is supported by the loss of BCG-induced effects in MΦ-depleted mice and in *Batf3^-/-^* mice lacking cross-presenting cDC1.^14^ Generally, TAM are considered tumoricidal when they express high levels of TNF, iNOS and MHC class II molecules (M1 MΦ), and pro-tumorigenic when they express high levels of arginase-1, IL-10, CD163 and CD204 (M2 MΦ).^41^ Here, we show that BCG lysate treatment enhanced the frequencies of M1-like tumoricidal, iNOS-positive TAM, suggesting a BCG lysate-mediated M1 polarization.

Preclinical studies have demonstrated that tumor immunity can be achieved by intratumoral drug administration, without the use of chemotherapy.^42 43^ However, systemic and intratumoral drug delivery have disadvantages, such as the need for multiple drug injections to maintain therapeutic levels and potential systemic adverse effects.^44^ Hydrogel-based drug delivery reduces the frequency of treatment, provides a more sustained immune response through gradual drug release and minimizes the risk of side effects.^26^ In our model, the thermosensitive BCG lysate-containing hydrogel was injected adjacent to the tumor and a single treatment was sufficient to induce tumor immunity, suppress metastasis and improve survival. Notably, BCG hydrogel treatment was associated with enhanced antigen processing and presentation in TDLN, promoting the induction of tumor-specific immune responses. Thus, the implantation of BCG hydrogel was more effective than intratumoral injections of BCG lysate in mediating long-lasting systemic immunity, suggesting that sustained drug release via hydrogel further improves the therapeutic efficacy of BCG lysate.

Despite the great progress in immunotherapy of malignant melanoma in the past decade, the treatment of disseminated unresectable metastases remains challenging. A prime cause of mortality in patients with melanoma is respiratory failure due to lung metastasis, as a consequence of therapy resistance or low targeting efficacy.^27 45^ In a metastatic melanoma model, BCG hydrogel significantly suppressed pulmonary metastasis. Previous studies have reported, that metastases show higher expressions of tumor-associated antigens (TAA) compared to primary lesions.^46^ TAA, such as TRP-2,^47^ and melanoma antigen reactive with T cells,^48^ can be recognized by TCRs of tumor-infiltrating CD8^+^ T lymphocytes, which contribute to the efficacy of most cancer immunotherapies.^49^ Therefore, characterization of the TCR repertoire and its antigen specificity within tumors is appropriate for assessing treatment efficacy. Here, we show that BCG hydrogel increased TCR diversity within tumors, which has been previously correlated with beneficial response to immune checkpoint blockade (ICB) therapy.^50^ In addition, the frequency of tumor-reactive CD8^+^ T cells was elevated in BCG hydrogel-treated tumors. Thus, BCG hydrogel could be an attractive adjunct to ICB therapies that together lead to increased activation of tumor-reactive and less auto-reactive T cells.^51–53^ Although altered TCR diversity can potentially explain tumor-directed immunity, the relationship between TCR diversity and TCR antigen specificity requires further investigation. Nevertheless, our findings indicate that BCG hydrogel treatment leads to increased TCR repertoire diversity and tumor antigen-specific TCR clones, resulting from enhanced CTSS expression and altered antigen processing and presentation in MΦ and DC. CTSS has been demonstrated to generate peptides for cross-priming of CD8^+^ T cells through MHC I antigen presentation.^30^ This is supported by our finding that BMDM and BMDC stimulated with BCG lysate showed increased CTSS expression as well as antigen cross-presentation to CD8^+^ T cells, which is abrogated by a CTSS inhibitor. The clinical relevance of these results is demonstrated by the fact that in human cutaneous melanoma biopsies, IL-BCG therapy was associated with enhanced M1 MΦ, mature DC, antigen processing and presentation, as well as with elevated CTSS expression. Moreover, the strong positive correlation of CTSS expression with improved survival in patients with melanoma suggests its potential as a prognostic biomarker.

In summary, our data imply several clinical benefits for the treatment of melanoma with BCG lysate-loaded hydrogel. First, BCG lysate is more potent than live BCG in tumor growth control and in the induction of a T cell inflamed, proinflammatory TME. Second, the use of BCG lysate in form of a hydrogel significantly enhanced survival and reduced the metastatic burden. Third, BCG hydrogel mediated CTSS-dependent antigen processing and presentation and led to enhanced TCR diversity with increased frequencies of tumor-specific CD8^+^ T lymphocytes. Finally, elevated CTSS expression was observed in melanoma biopsies on intralesional BCG therapy, and positively correlated with improved survival in patients with melanoma. Overall, our results provide insight into the mechanisms and a rationale for the clinical translation of BCG hydrogel as an effective and safe immunotherapeutic option for the treatment of melanoma.

10.1136/jitc-2021-004133.supp2 Supplementary data

## Data Availability

Data are available upon reasonable request.
