# Supplementary material for: BCG hydrogel promotes CTSS-mediated antigen processing and presentation, thereby suppressing metastasis and prolonging survival in melanoma
Source: J Immunother Cancer. 2022 Jun 22;10(6):e004133. doi: 10.1136/jitc-2021-004133 (PMC9226922; doi:10.1136/jitc-2021-004133)
Supplement: Supplementary data [file jitc-2021-004133supp001.pdf]

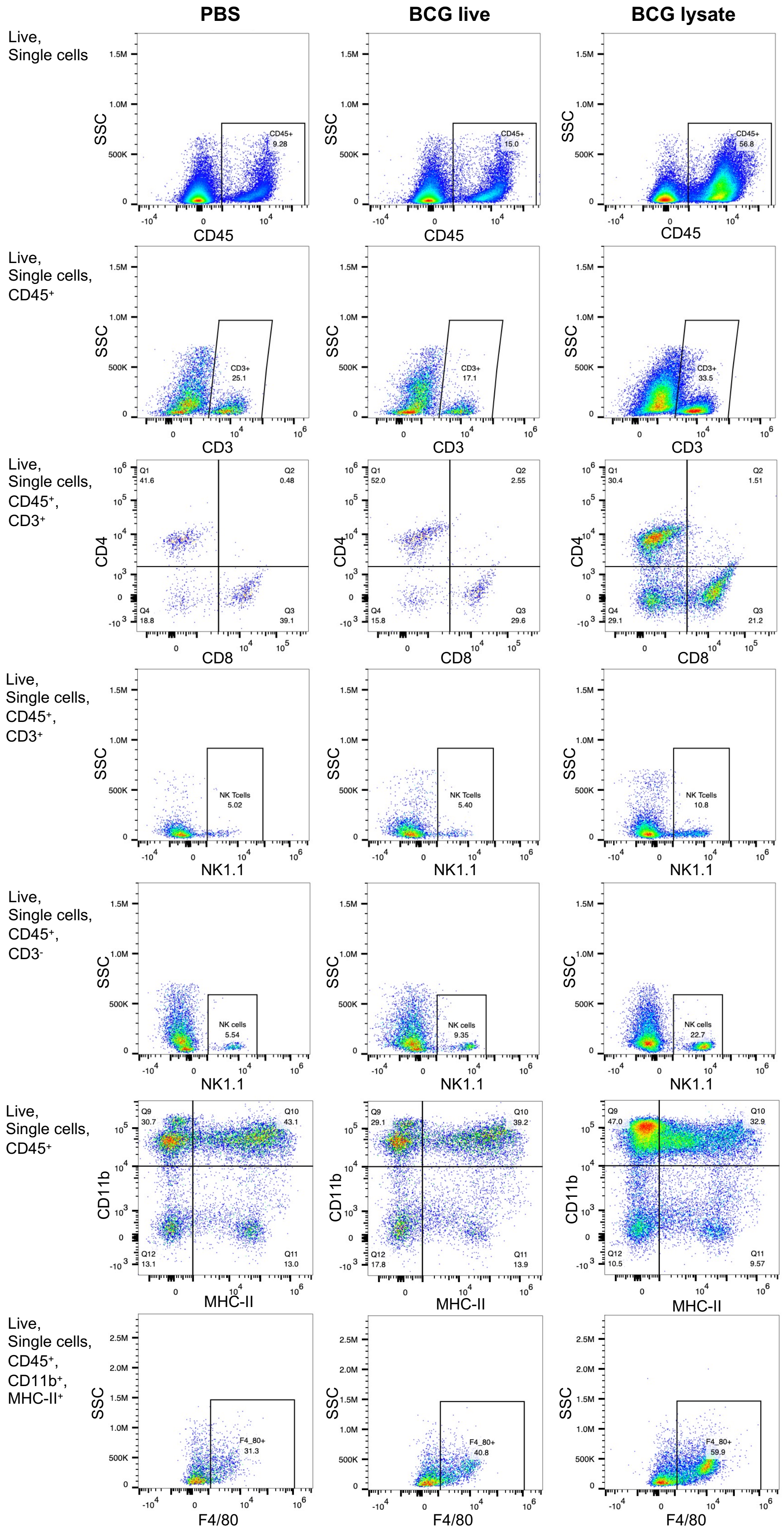

Supplemental Figure S1. BCG lysate treatment enhances immune cell infiltration. C57BL/6J wt mice were injected with $2x10^5$ B16F10 melanoma cells and treated with live BCG, BCG lysate or PBS on day 7 and 11 after tumor injection. Representative flow cytometry plots displaying gating strategies for major immune cell subsets in each treatment group are shown.

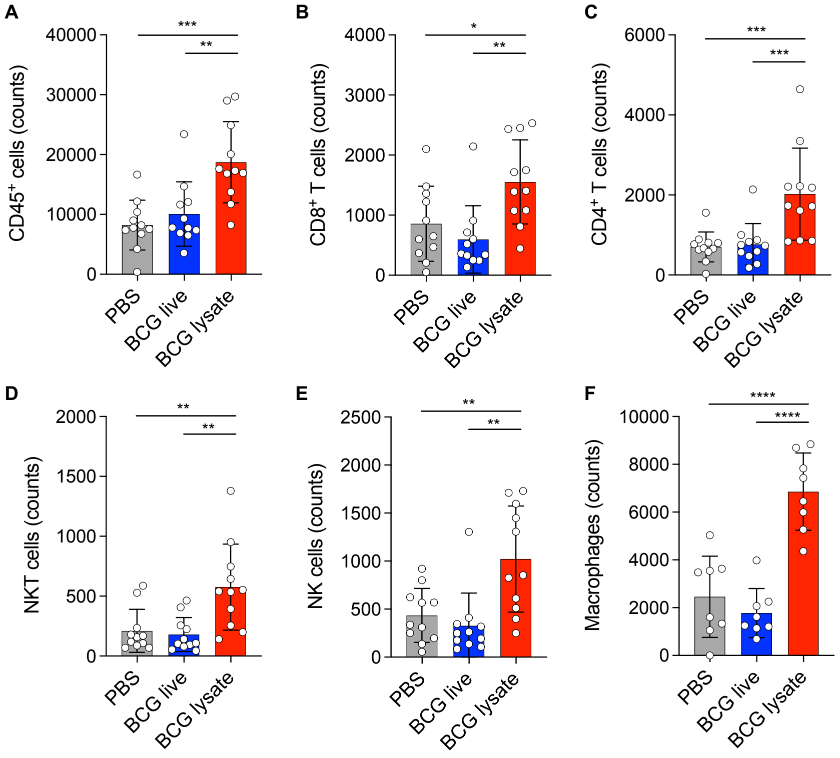

Supplemental Figure S2. BCG lysate treatment enhances immune cell infiltration. (**A-F**) C57BL/6J wt mice were injected with $2x10^5$ B16F10 melanoma cells and treated with live BCG, BCG lysate or PBS on day 7 and 11 after tumor injection. Absolute cell counts of cell subsets of (**A**) $CD45^+$ cells, (**B**) $CD8^+$ T cells, (**C**) $CD4^+$ T cells, (**D**) NKT cells, (**E**) NK cells (n=11 per group, two independent experiments) and (**F**) macrophages (n=8 per group) measured by flow cytometry. Data are shown as mean ± SD using One-way ANOVA, followed by Tukey's multiple comparisons test. P ≤ 0.0001 (****).

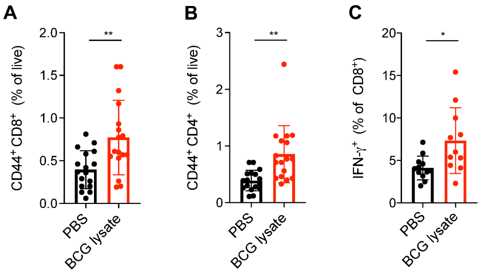

Supplemental Figure S3: BCG lysate-treated tumors show enhanced T cell activation. (**A**-**C**) C57BL/6J wt mice were injected with 2x10^5 B16F10 melanoma cells and treated with BCG lysate or PBS on day 7 and 11 after tumor injection. Relative frequencies of (**A**) $CD44^+CD8^+$ T cells and (**B**) $CD44^+CD4^+$ T cells and (n=17 per group, three independent experiments), (**C**) IFN-γ^+ $CD8^+$ T cells (n=12 per group, two independent experiments) measured by flow cytometry are shown as mean ± SD using unpaired, 2-tailed Student's *t* test P ≤ 0.05 (*), P ≤ 0.01 (**).

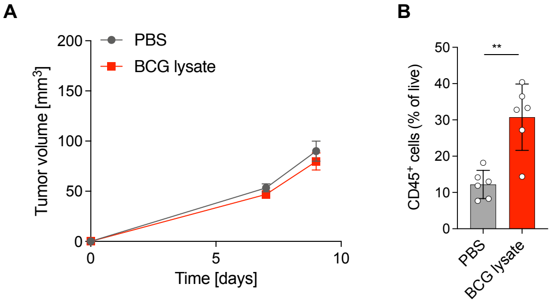

Supplemental Figure S4: B16F10 melanomas treated with BCG lysate already show increased infiltration of immune cells at early timepoints. (**A,B**) C57BL/6J wt mice were injected with $2x10^5$ B16F10 melanoma cells and treated with BCG lysate or PBS on day 7 after tumor injection. Tumors were isolated on day 9, and (**A**) growth curve and (**B**) flow cytometric analysis of $CD45^+$ immune cell infiltration of treated and untreated tumors were assessed (n=6 per group). Data are shown as mean relative frequencies ± SD using unpaired, 2-tailed Student's *t* test, P ≤ 0.01 (**).

Kremenovic M, *et al. J Immunother Cancer* 2022; 10:e004133. doi: 10.1136/jitc-2021-004133

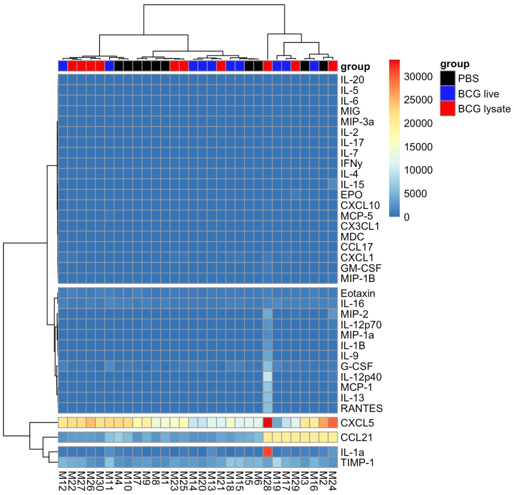

Supplemental Figure S5: BCG treatment did not induce systemic inflammation in tumor-bearing mice. Heatmap showing protein expression levels of cytokines and chemokines in sera of mice treated with live BCG or BCG lysate on day 12 post tumor inoculation, 24h after the last treatment (n=10 per group).

Kremenovic M, *et al. J Immunother Cancer* 2022; 10:e004133. doi: 10.1136/jitc-2021-004133

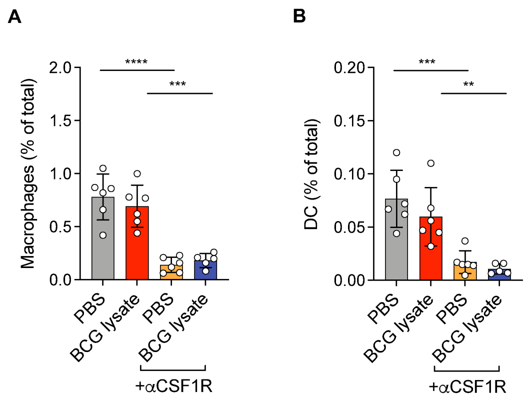

Supplemental Figure S6. Depletion of splenic macrophages and DC using anti-CSF1R. Relative frequencies of macrophages (**A**) and DC (**B**) measured by flow cytometry in the spleen. Data are represented as mean ± SD using One-way ANOVA followed by Tukey's multiple comparisons test (n=5-6 per group). Data are shown as mean ± SD using One-way ANOVA, followed by Tukey's multiple comparisons test. P ≤ 0.01 (**), P ≤ 0.001 (***), P ≤ 0.0001 (****).

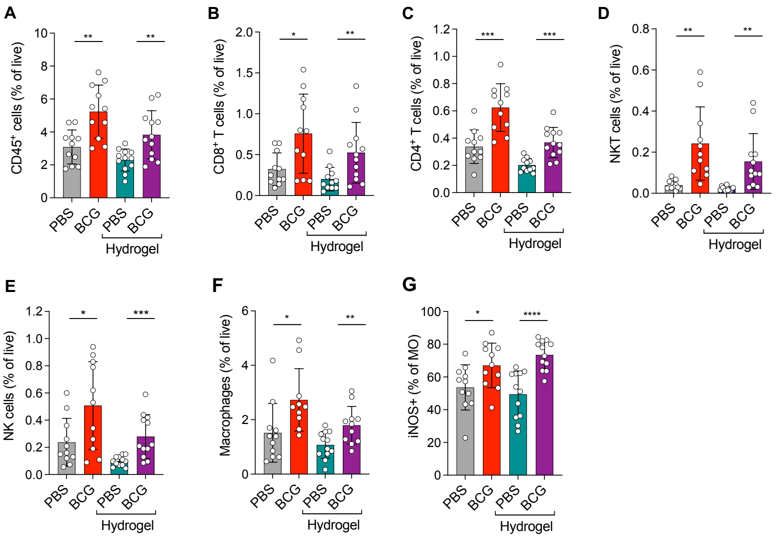

Supplemental Figure S7: BCG hydrogel treatment promotes immune infiltration into the tumor. (**A-G**) Relative frequencies of cell subsets in primary, treated tumors measured by flow cytometry: (**A**) $CD45^+$ cells, (**B**) $CD8^+$ T cells, (**C**) $CD4^+$ T cells**,** (**D**) NKT cells, (**E**) NK cells, (**F**) macrophages and (**G**) $iNOS^+$ macrophages. Data are shown as mean ± SD using unpaired, 2-tailed Student's *t* test (n=12 per group, two independent experiments). P ≤ 0.05 (*), P ≤ 0.01 (**), P ≤ 0.001 (***), P ≤ 0.0001 (****).

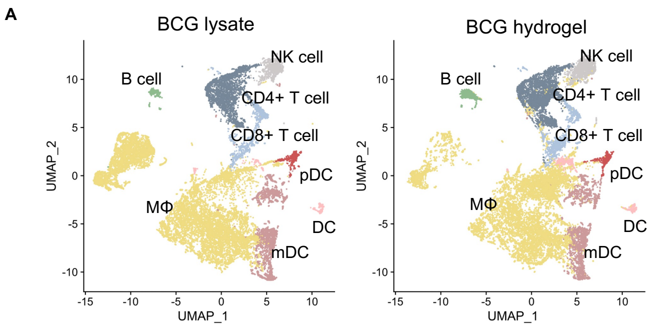

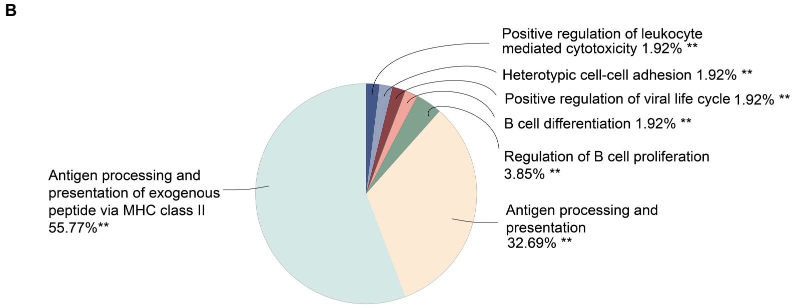

Supplemental Figure S8: BCG hydrogel promotes DC maturation and antigen processing and presentation. ScRNA-Seq analysis of tumors treated with BCG lysate or BCG hydrogel. (**A**) UMAP dimensional reduction of BCG lysate and BCG hydrogel-treated tumors. (**B**), Gene ontology analysis of biological processes for genes upregulated in BCG hydrogel-treated tumors compared to BCG lysate-treated tumors. Differentially upregulated genes were identified using $\log_2$ fold change cut-off = 0.25 and FDR = 0.05.

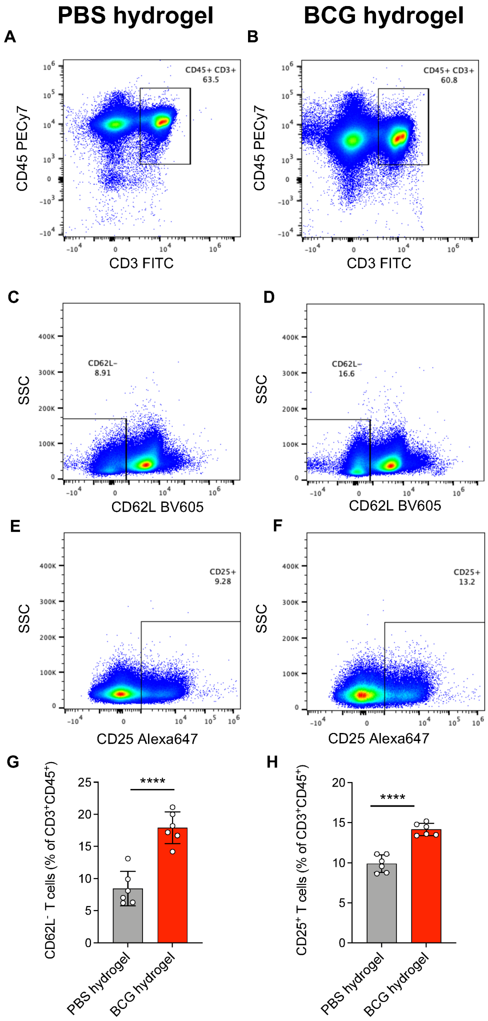

Supplemental Figure S9: Relative frequencies of activated T cells in tumor-draining lymph nodes from (**A, C, E**) PBS hydrogel and (**B, D, F**) BCG hydrogel treated tumor-bearing mice measured by flow cytometry. Representative FACS panels gated on (**A, B**) $CD45^+CD3^+$ lymphocytes, (**C, D**) $CD62L^-$ and (**E, F**) $CD25^+$ activated T cells are shown. Relative frequencies of (**G**) $CD62L^-$ and (**H**) $CD25^+$ of $CD45^+CD3^+$ cells are shown as mean ± SD using unpaired, 2-tailed Student's *t* test (n=6). P ≤ 0.0001 (****).

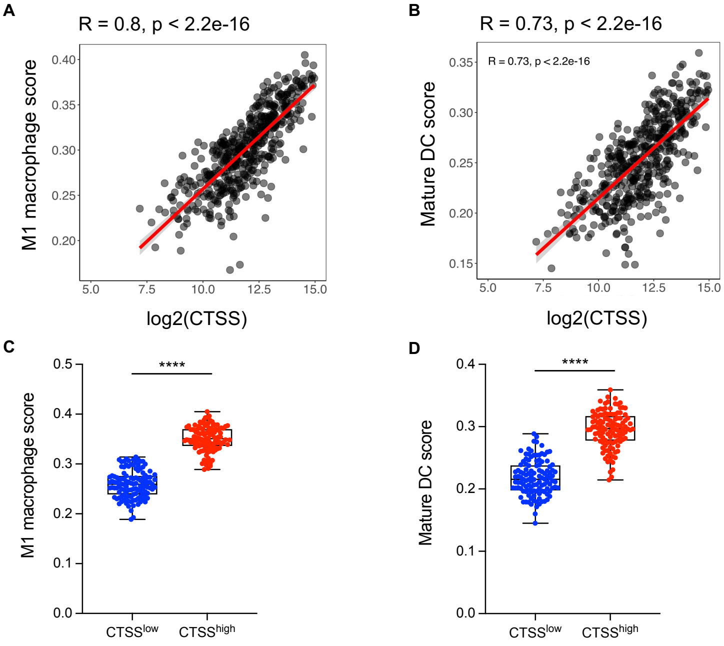

Supplemental Figure S10: CTSS expression positively correlates with M1 macrophages and mature DC in SKCM patients. (**A, B**) Pearson correlation between *CTSS* mRNA expression and (**A**) M1 macrophage and (**B**) mature DC score in SKCM cohort. (**C**) M1 macrophage and (**D**) mature DC score in $CTSS^{high}$ and $CTSS^{low}$ expressing SKCM patients (bottom and top 25%,n = 118). The box extends between 25% and 75%, and the whisker extends up to 75% plus IQR and down to 25% minus IQR. Statistical analysis was performed by unpaired, 2-tailed Student's t test. P ≤ 0.0001 (****).

Kremenovic M, *et al. J Immunother Cancer* 2022; 10:e004133. doi: 10.1136/jitc-2021-004133

Supplemental Table S1: Gene signatures for M1 macrophages (Becker M *et al*. *Sci Rep*, 2015), mature DC (Jin P *et al*. *J Transl Med*, 2010) and antigen processing and presentation (Kanehisa M, *Nucleic Acids Res*, 2000).

**M1 macrophage**		**Mature DC**	**Antigen processing and presentation**	
ADAM28	LIMK2	CCL5	B2M	KIR2DS4
AIM2	LRRK2	CXCL10	CALR	KIR2DS5
ANKRD22	MUC1	CCR7	CANX	KIR3DL1
APOBEC3A	MX1	IL15	CD4	KIR3DL2
APOL1	NAMPT	IFI27	CD74	KIR3DL3
APOL3	NFKBIZ	IFI44L	CD8A	KLRC1
BATF2	OAS1	IFIH1	CD8B	KLRC2
C1R	OAS2	IFIT1	CIITA	KLRC3
C1S	OAS3	MX1	CREB1	KLRC4
CCL19	OASL	ISG15	CTSB	KLRD1
CD38	OPTN	ISG20	CTSS	LGMN
CD40	PAG1	IRF7	HLA-A	LTA
CD80	PARP14	GBP4	HLA-B	NFYA
CFB	PCNX	DUSP5	HLA-C	NFYB
CLEC4D	PDE4B	NFKBIA	HLA-DMA	NFYC
CXCL10	PIM1	ATF3	HLA-DMB	PDIA3
CXCL9	PRKAR2B	TNFSF10	HLA-DOA	PSME1
CYBB	PSMB9	IL6	HLA-DOB	PSME2
DUSP10	PTGS2	CXCL8	HLA-DPA1	RFX5
DUSP6	RARRES3	IL7R	HLA-DPB1	RFXANK
ETV7	RCN1	CCL4	HLA-DQA1	RFXAP
FAM49A	RHBDF2	TNFAIP6	HLA-DQA2	TAP1
FAM65B	RSAD2	IFIT3	HLA-DQB1	TAP2
FCGR1B	SAT1	OASL	HLA-DQB2	TAPBP
FPR2	SCO2	GBP1	HLA-DRA	
GADD45G	SERPING1	HES4	HLA-DRB1	
GBP1	SLAMF7	CYP27B1	HLA-DRB3	
GBP2	SLC22A15	RIPK2	HLA-DRB4	
GBP4	SLC25A28	TNFRSF9	HLA-DRB5	
GBP5	SLC31A2	SOD2	HLA-E	
GCH1	SLC6A12	CD38	HLA-F	
GK	SLC7A5	CD44	HLA-G	
GPR84	SNTB1	CD80	HSP90AA1	
GUCY1A3	SNX10	CD83	HSP90AB1	
HERC5	SOCS3	CD86	HSPA5	
HESX1	SOD2	IDO1	IFI30	
HLA-F	STAT1	MT2A	IFNA1	
IFI27	STAT3	TRAF1	IFNA10	
IFI35	STX11	GADD45B	IFNA13	
IFI44L	TAP1	MT1M	IFNA14	
IFIH1	TNFAIP6	MT1HL1	IFNA16	
IFIT2	TNFSF10	BIRC3	IFNA17	
IFIT3	TRIM69	USP18	IFNA2	
IFITM1	UBE2L6	TUBB2A	IFNA21	
IFITM2	USP18	CCL8	IFNA4	
IL15	VAMP5	EBI3	IFNA5	
IL15RA	WARS	IFITM1	IFNA6	
IL32	XRN1	MT1B	IFNA7	
INHBA		MT1E	IFNA8	
IRF1		MT1G	KIR2DL1	
IRF7		MT1H	KIR2DL2	
ISG15		GADD45A	KIR2DL3	
ISG20		CD200	KIR2DL4	
ITGAL		LAMP3	KIR2DL5A	
ITGB7		RGS1	KIR2DS1	
LAG3		SAT1	KIR2DS2	
LAMP3			KIR2DS3	
