# Supplementary material for: BCG hydrogel promotes CTSS-mediated antigen processing and presentation, thereby suppressing metastasis and prolonging survival in melanoma
Source: J Immunother Cancer. 2022 Jun 22;10(6):e004133. doi: 10.1136/jitc-2021-004133 (PMC9226922; doi:10.1136/jitc-2021-004133)
Supplement: Supplementary data [file jitc-2021-004133supp002.pdf]

# BCG hydrogel promotes CTSS-mediated antigen processing and presentation, thereby suppressing metastasis and prolonging survival in melanoma

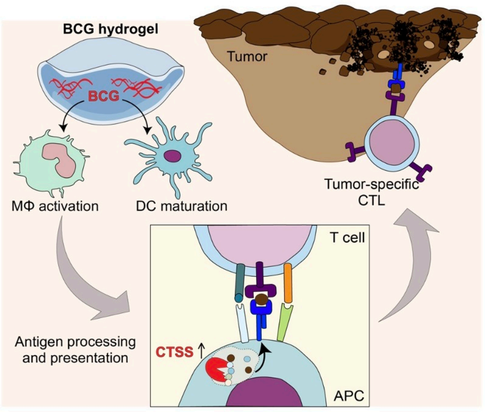


**Authors**
Mirela Kremenovic, Alfred A. Chan, Bing Feng, Lukas Bäriswyl, Steve Robatel, Thomas Gruber, Li Tang, Delphine J. Lee, Mirjam Schenk.

**Correspondence**
mirjam.schenk@pathology.unibe.ch

**In Brief**
We are the first to formulate a BCG lysate-containing hydrogel (BCG hydrogel) that reduces the metastatic potential of B16F10 melanoma and increases survival of tumor-bearing mice. BCG hydrogel promotes cathepsin S (CTSS) activity in macrophages and dendritic cells, resulting in enhanced antigen processing and presentation of tumor-associated antigens.
